# Gangrenous cecal volvulus with 720° torsion and prolonged intraoperative hypotension in a resource-limited setting: a case report

**DOI:** 10.1097/MS9.0000000000005272

**Published:** 2026-06-18

**Authors:** Amanuel M. Oljira, Boka I. Shoro, Abebe M. Tebelu, Bisrat A. Bekele, Dagem A. Gebremichael

**Affiliations:** a Department of Surgery, Ambo University College of Medicine and Health Science, Ambo, Ethiopia; b Department of Public Health, School of Medicine, Addis Ababa University College of Health Science, Addis Ababa, Ethiopia

**Keywords:** case report, cecal volvulus, gangrene, intraoperative hypotension, resource-limited setting, staged diversion

## Abstract

**Introduction and importance:**

Cecal volvulus is an uncommon cause of large-bowel obstruction, but when it occurs, it can rapidly become life-threatening, with ischemia and gangrene developing quickly. Explicitly quantified 720° torsion is rarely described.

**Case presentation:**

A 45-year-old man presented after 5 days of abdominal pain, distension, obstipation, fever, and vomiting. His pulse rate was 124 beats per minute, his blood pressure was 100/65 mmHg, and he demonstrated clear signs of peritonism. Based on his history, examination findings, elevated white blood cell count, and abdominal X-ray, we suspected a gangrenous large-bowel obstruction. During laparotomy, we found the cecum and ascending colon twisted 720° counterclockwise, with gangrene extending up to the hepatic flexure and involving the distal ileum. The right colon was mobilized lateral-to-medial, the ileocolic vessels were ligated, and the mesenteric division was completed using hand-tied 3-0 Vicryl ligatures. Bowel transection was performed in healthy tissue approximately 10 cm proximal to the ileocecal valve, followed by en bloc resection of the gangrenous cecum and ascending colon. He remained hypotensive intraoperatively for about 40 minutes, with a nadir of 79/51 mmHg, and required crystalloids along with norepinephrine at 0.05 μg/kg/min. Owing to his instability, an end ileostomy and proximal transverse colonic mucous fistula were created instead of a primary anastomosis.

**Clinical discussion:**

This case highlights that reconstruction in gangrenous cecal volvulus should be individualized according to bowel viability, contamination, and patient physiology. In the presence of prolonged intraoperative hypotension, vasopressor requirement, bowel edema, and severe bowel compromise, staged diversion may be safer than immediate anastomosis. The case also emphasizes the diagnostic limitations of plain radiography in distinguishing cecal from sigmoid volvulus when CT is unavailable.

**Conclusion:**

This case illustrates that urgent source control and physiology-guided staged reconstruction can achieve good outcomes in severe cecal volvulus, even in a resource-limited referral hospital without CT or pathology services.

## Introduction

Cecal volvulus is a rare condition, but when it occurs, ischemia, gangrene, and even perforation can develop rapidly if not managed promptly^[^1^]^. Surgery is necessary, especially when the bowel is compromised, and reconstruction should be planned based on the patient’s stability and operative risk^[^2^]^. Outcomes are worse when gangrene, peritonitis, or physiologic instability are present^[^3^]^. CT scans are helpful for diagnosis when available^[^4^]^, but in many areas, delayed presentation or lack of imaging worsens management and results^[^5^]^. Endoscopic decompression generally does not work for cecal volvulus^[^6^]^. This case presents a severe 720° gangrenous cecal volvulus with prolonged intraoperative hypotension requiring norepinephrine, managed with staged diversion and delayed restoration of bowel continuity in a setting without CT, colonoscopy, or pathology services. This case report is reported in accordance with the SCARE 2023 criteria^[^7^]^. The key message is that bowel reconstruction in gangrenous cecal volvulus should be individualized according to physiology, bowel viability, and operative risk, whether in a high-resource hospital or a resource-limited environment.

HIGHLIGHTS

- Gangrenous cecal volvulus with explicitly quantified 720° torsion is an unusually severe presentation and is rarely described in the literature.
- Prolonged intraoperative hypotension, vasopressor requirement, bowel edema, and severe bowel compromise prompted physiology-guided staged diversion rather than immediate anastomosis.
- Prompt laparotomy, en bloc resection, and delayed restoration of bowel continuity resulted in a favorable 1-year outcome despite the absence of CT, colonoscopy, and pathology services.

## Case presentation

The patient was managed at a referral hospital in a resource-limited setting where CT imaging, colonoscopy, and pathology services were unavailable. A 45-year-old man, weighing 62 kg (BMI 21 kg/m^2^), who worked as a farmer, was referred from a lower-level facility to our referral hospital after 5 days of worsening abdominal pain, increasing bloating, complete constipation, fever, and repeated vomiting. He had no history of abdominal surgery or other health issues. He was not taking regular medications, had no known drug allergies, and had no relevant family history. He did not smoke and reported no alcohol or substance use.

At presentation, his vital signs were: blood pressure 100/65 mmHg, heart rate 124 beats/min, respiratory rate 22 breaths/min, and temperature 38.1°C. His abdomen was very distended, diffusely tender, with rebound tenderness and involuntary guarding. The rectal exam showed an empty rectum.

**Figure 1.**
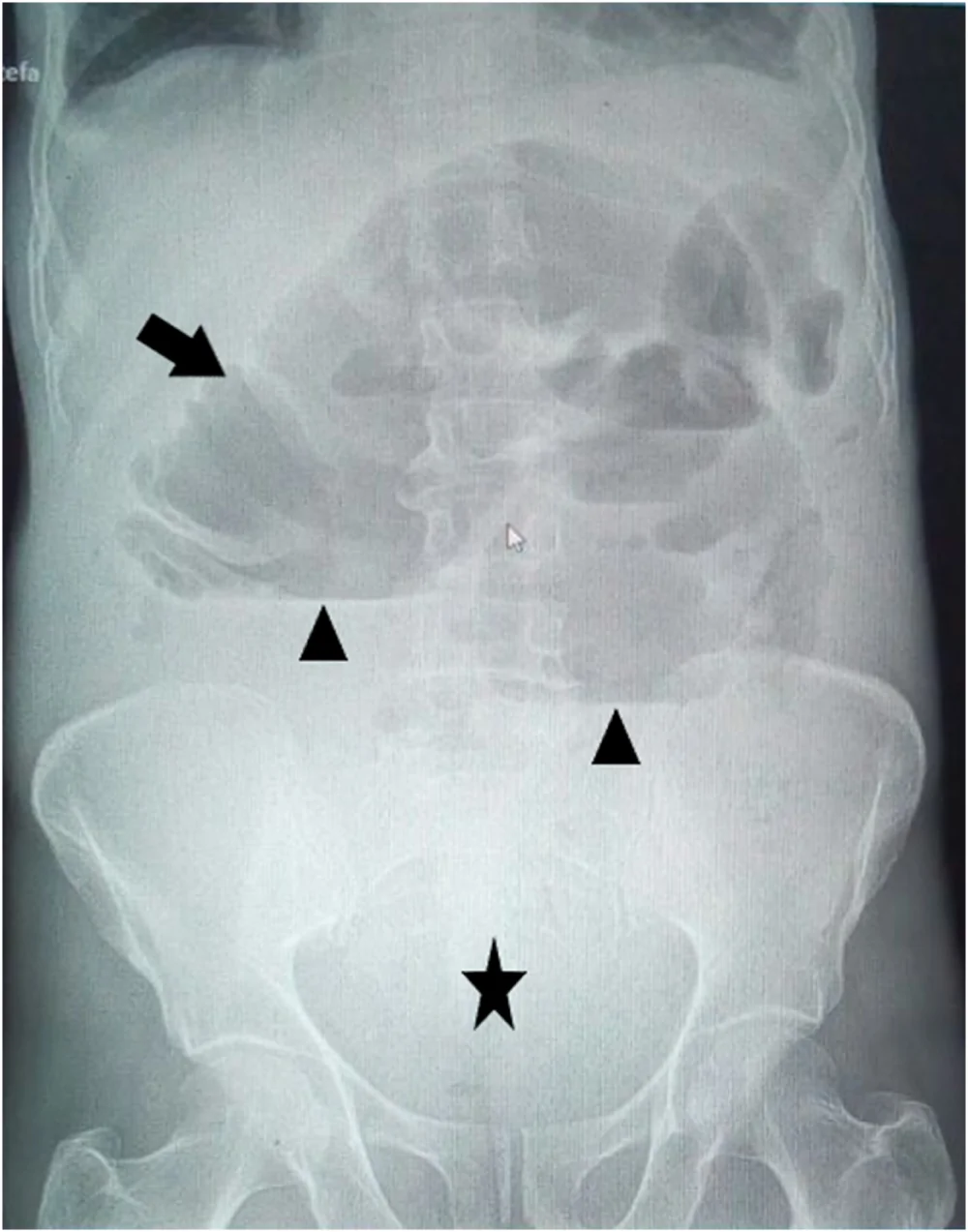
Erect anteroposterior (AP) abdominal radiograph demonstrating findings suggestive of cecal volvulus. The solid black arrow indicates the massively distended, ectopic cecum displacing into the mid-to-left upper abdomen with notable preservation of the haustral folds. The black arrowheads point to prominent, distinct air-fluid levels within the dependent portions of the obstructed bowel loops, and rectal gas paucity is indicated by the black star.

Laboratory tests showed a white blood cell count of 14.0 × 10^3^/µL with 89% neutrophils, hemoglobin 14.0 g/dL, platelets 241 × 10^3^/µL, and normal electrolytes. An abdominal X-ray revealed dilated large-bowel loops and air–fluid levels, consistent with large-bowel obstruction. (Fig. 1) CT and colonoscopy were not available.

Preoperative assessment: Based on his history (5 days of worsening bloating, constipation, fever, and vomiting), physical findings (diffuse tenderness, rebound, and guarding), laboratory results (elevated white blood cell count and neutrophilia), and abdominal X-ray, we suspected a strangulating or gangrenous large-bowel obstruction, possibly with ischemia or perforation. We also considered other possibilities – sigmoid volvulus, obstructing colon cancer, pseudo-obstruction, severe appendicitis, and adhesive small-bowel obstruction. Still, the combination of radiographic findings, peritonism, and systemic toxicity strongly suggested a gangrenous or strangulating obstruction, so we proceeded rapidly to surgery for a definitive diagnosis and treatment.

CT and colonoscopy were not available, and transfer for advanced imaging would only have delayed surgery and source control. Before surgery, the patient underwent rapid resuscitation with intravenous crystalloids (2 L lactated Ringer’s bolus), nil per os, analgesia with tramadol 50 mg intravenously three times daily, nasogastric decompression, urinary catheterization, and broad-spectrum intravenous antibiotics (ceftriaxone 1 g IV and metronidazole 500 mg IV). We then proceeded directly to urgent laparotomy, confirming the diagnosis of gangrenous cecal volvulus.

**Figure 2.**
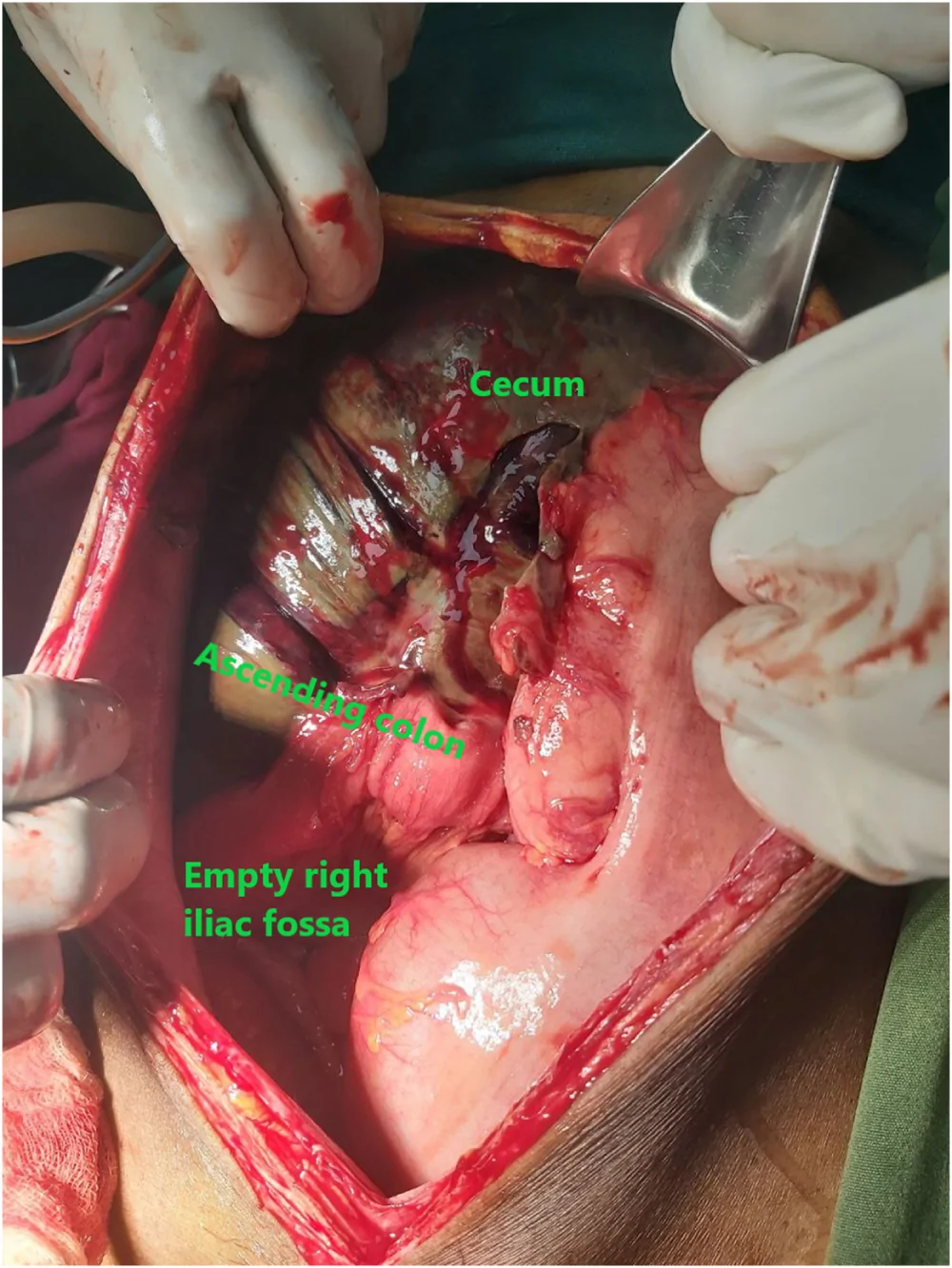
Intraoperative photograph during exploratory laparotomy demonstrating a gangrenous cecal volvulus. The cecum is massively distended and shows features of transmural necrosis (dusky black discoloration). The ascending colon is involved in the torsion site. Note the empty right iliac fossa, a key diagnostic finding indicating migration of the hypermobile cecum from its normal anatomical position in the right lower quadrant.

Under general anesthesia, the patient was placed in the supine position, and the abdomen was prepared and draped in the standard sterile fashion. Intraoperatively, 500 mL of dark, reactive peritoneal fluid was found. The cecum and ascending colon were completely mobile and twisted 720° counterclockwise, lying in the right upper quadrant. (Fig. 2) Gangrene extended up to the hepatic flexure and involved about 10 cm of terminal ileum, but there was no obvious fecal contamination. The operation was led by a general surgeon experienced in emergency gastrointestinal surgery. The right colon was mobilized using a lateral-to-medial approach by incising the white line of Toldt. The right ureter and the second part of the duodenum were identified and carefully preserved. Vascular control was achieved by isolated ligation of the ileocolic vessels. Mesenteric division was completed using hand-tied 3-0 Vicryl suture ligatures to secure the mesenteric pedicles and associated small vessels. Bowel transection was performed in healthy, well-perfused tissue, with the ileal resection margin approximately 10 cm proximal to the ileocecal valve. The gangrenous cecum and ascending colon were resected en bloc. Estimated blood loss was 150 mL. The total operative duration was 2 hours and 25 minutes.

During resection, the patient’s blood pressure dropped and remained low for around 40 minutes, fluctuating between 79/51 and 88/50 mmHg, while the heart rate increased. The team promptly administered additional balanced crystalloid boluses as necessary and started norepinephrine at 0.05 μg/kg/min. They also clamped the gangrenous bowel and suctioned out reactive fluid. After about 45 minutes, the blood pressure improved to 98/65 mmHg, and the heart rate decreased to 108 beats/min.

The decision to defer primary anastomosis was guided by a physiology-based intraoperative risk assessment. In addition to prolonged intraoperative hypotension and vasopressor requirement, the patient had advanced disease with peritonism at presentation, gangrenous bowel, bowel edema, reactive intraperitoneal fluid, and overall physiologic derangement. These factors raised concern regarding impaired bowel perfusion and an unacceptably high anastomotic risk at the time of reconstruction. The operative strategy was therefore conceptualized as physiology-guided staged reconstruction and anastomotic risk mitigation, with overlap with damage-control principles, rather than as a formal abbreviated damage-control laparotomy. The proximal ileal end was brought out through the right lower quadrant as an end ileostomy. The distal bowel end, consisting of the proximal transverse colon, was matured as a mucous fistula in the upper abdomen. Both stomas were matured using interrupted 3-0 absorbable sutures. After warm saline irrigation and confirmation of hemostasis, the abdominal fascia was closed in a standard fashion.

Histopathologic examination was not performed because pathology services were unavailable locally, and no nearby referral pathology service was accessible. Therefore, the resected specimen was not submitted for pathologic analysis. Intraoperatively, gangrene was diagnosed by gross assessment of the involved terminal ileum, cecum, and ascending colon. Findings included transmural dusky-green discoloration, complete loss of serosal sheen, absence of mural bleeding on mesenteric incision, bowel wall thinning and friability, and absent peristalsis despite warm saline application, consistent with irreversible ischemic necrosis.

## Timeline

**Day 0:** Presentation, resuscitation, plain radiography, emergency laparotomy, en bloc resection, end ileostomy, and proximal transverse colonic mucous fistula.

**Postoperative day 2:** Stoma functioning and recovery progressing satisfactorily. **Postoperative month 3:** Stoma reversal through the same incision with a two-layer hand-sewn end-to-side ileotransverse anastomosis.

**Post-reversal day 5:** Discharged without complications.

**12-month follow-up**: No recurrence, no stoma-related sequelae, and full return to normal activity.

Postoperatively, the patient was managed on the surgical ward with intravenous fluids (calculated maintenance lactated Ringer’s solution), electrolyte monitoring, analgesia (diclofenac 75 mg intramuscularly twice daily for 3 days, followed by paracetamol 1 g orally four times daily after bowel function returned), thromboprophylaxis (unfractionated heparin 7500 IU subcutaneously twice daily), antibiotic therapy (ceftriaxone 1 g IV twice daily and metronidazole 500 mg IV three times daily), and stoma-care education. Postoperative recovery was uneventful. The stoma began functioning within 36 hours. The ileostomy output ranged from 650 to 900 mL/day; there were no signs of dehydration or renal dysfunction; and serum creatinine remained 0.7–1.0 mg/dL. The patient said, “I was scared because my pain and vomiting kept worsening, and my abdomen became very swollen. After the first surgery, adjusting to the stomas was difficult at first, but with teaching, I managed them well.”

Three months later, stoma reversal was performed through the same incision using a two-layer hand-sewn end-to-side ileotransverse anastomosis after standard antibiotics. The patient experienced no complications and was discharged on postoperative day 5. No postoperative complications occurred after either procedure, and no Clavien–Dindo grade I–V events were observed. One year after surgery, he had resumed normal life without any complications.

## Discussion

This patient presented late, already displaying peritonism and systemic upset, so the team quickly proceeded to surgery. CT imaging can reveal the classic features of cecal volvulus and potential complications^[^4^]^, but in many hospitals, waiting for imaging or transferring the patient causes delays, which worsen outcomes, especially in resource-limited settings^[^5^]^. Endoscopic decompression is not recommended for cecal volvulus^[^6^]^, so surgery is indicated when ischemia is suspected. In this case, the diagnosis was gangrenous large-bowel obstruction preoperatively, based on clinical and radiographic findings. The diagnosis of gangrenous cecal volvulus was confirmed intraoperatively.

Because CT was unavailable, the diagnosis relied on clinical findings and plain radiography. Plain abdominal radiographs are useful as an initial test in bowel obstruction but are less sensitive for cecal volvulus than for sigmoid volvulus^[^4,8^]^. Sigmoid volvulus more often shows disproportionate sigmoid dilatation, absent rectal gas, and classic signs such as the northern exposure sign, Frimann-Dahl sign, and liver overlap sign, whereas cecal volvulus may show a markedly dilated ectopic cecum, a single large air-fluid level, and sometimes preserved haustral markings^[^8^]^. However, many cecal volvulus cases lack specific radiographic features. In our patient, radiography supported the diagnosis of large-bowel obstruction but could not definitively distinguish cecal from sigmoid volvulus preoperatively.

The operative management of cecal volvulus primarily depends on the viability of the bowel and the patient’s condition. In viable cecal volvulus, detorsion with fixation procedures, such as cecopexy, has been described, though recurrence remains a major concern^[^2,9^]^. Once gangrene is present, resection is mandatory^[^2,3^]^. In a physiologically stable patient with viable resection margins and acceptable contamination, right hemicolectomy or ileocolic resection with primary anastomosis is often appropriate^[^2,9^]^. In contrast, resection with stoma creation or staged reconstruction is preferable when the patient is unstable, the bowel is edematous, contamination is significant, or tissue perfusion is questionable^[^2,3^]^.

A key intraoperative issue in this case was the prolonged hypotension during resection. Current perioperative literature emphasizes that both the depth and duration of intraoperative hypotension are associated with adverse postoperative outcomes^[^10,11^]^. In colorectal surgery, vasopressor use may reflect underlying physiologic instability and has been associated with concerns regarding impaired anastomotic perfusion, particularly when coupled with hypovolemia, bowel edema, or sepsis-related vasodilation^[^12^]^. In our case, the decision to avoid immediate anastomosis was therefore not based on vasopressor use alone but on a physiology-based intraoperative risk assessment incorporating bowel perfusion, bowel edema, peritonism, reactive intraperitoneal fluid, and severe bowel compromise. The strategy is best understood as physiology-guided staged reconstruction and anastomotic risk mitigation, rather than as a broad practice-changing recommendation. This interpretation is also consistent with damage-control principles that prioritize source control and physiologic recovery before definitive reconstruction in selected unstable patients^[^13^]^.

The degree of torsion is not consistently quantified in volvulus reports, which limits direct comparison across the literature. In a focused literature search, reports of 720° torsion were identified in total colonic volvulus and transverse colon volvulus, with favorable postoperative outcomes^[^14,15^]^. In contrast, most published cecal volvulus reports describe torsion and bowel compromise without clearly specifying the exact angle of rotation^[^9^]^. We therefore regard the present case not as definitively unique but as an unusually severe and explicitly quantified 720° gangrenous cecal volvulus managed successfully with staged reconstruction in a resource-limited environment.

Our patient’s postoperative course was favorable. Ileostomy output remained within an acceptable range without renal compromise, and delayed restoration of continuity was successful at 3 months, followed by an uneventful 1-year follow-up^[^16^]^. This outcome supports the appropriateness of a staged approach in this specific physiologic context.

A limitation of this report is the absence of histopathologic confirmation. Because pathology services were unavailable, gangrene was diagnosed solely by gross intraoperative assessment. Although the macroscopic findings were strongly consistent with irreversible ischemic necrosis, alternative underlying pathology, such as occult malignancy, cannot be excluded with absolute certainty. However, no gross intraoperative findings suggested a neoplastic cause, and the operative findings were most consistent with gangrenous cecal volvulus. Another limitation is the absence of preoperative CT imaging, which prevented definitive radiologic subtyping before laparotomy. A strength of this report is the detailed intraoperative documentation of the degree of torsion, bowel involvement, and physiology-guided reconstructive decision-making.

## Conclusion

This case illustrates that gangrenous cecal volvulus requires urgent operative source control and that bowel reconstruction should be individualized according to intraoperative physiology. In this patient, prolonged hypotension, vasopressor requirement, bowel edema, and severe bowel compromise favored staged diversion over immediate anastomosis. Even in a resource-limited referral hospital without CT or pathology services, prompt surgery and physiology-guided decision-making resulted in successful delayed restoration of bowel continuity and a good 1-year outcome.

## Key takeaway message

- Preoperatively, a diagnosis of gangrenous/strangulating large-bowel obstruction can be justified using history, signs of peritonitis on examination, leukocytosis, and plain radiography when advanced imaging is unavailable.
- Cecal volvulus may be confirmed intraoperatively; timely laparotomy remains decisive when ischemia is suspected.
- When prolonged intraoperative hypotension and vasopressor support are required, diversion-first with delayed anastomosis may be a reasonable strategy in selected unstable patients with a high anastomotic risk.

## Data Availability

Not applicable. All relevant clinical data are included within the article.

## Research registration unique identifying number (UIN)

Not applicable for a single case report; no prospective research registration is required.

## Assistance with the study

The authors would like to thank the Emergency Department team for the initial resuscitation and coordination of urgent care, and the Operating Theater staff for their support during emergency surgery and perioperative management. We confirm that no individuals are named, and written permission would be obtained if any individual were acknowledged by name.

## Reporting guideline compliance

This surgical case report has been reported in line with the SCARE 2025 criteria. A completed SCARE checklist is uploaded as Supplemental Digital Content Material, available at: http://links.lww.com/MS9/B267, as required.

## AI transparency

No AI tools were used in writing this manuscript, producing figures/graphics, or collecting/analyzing data. A completed TITAN checklist declaring no AI use is uploaded as Supplemental Digital Content Material, available at: http://links.lww.com/MS9/B267, as required.
